# Crustal anisotropy across northern Japan from receiver functions

**DOI:** 10.1002/2014JB011681

**Published:** 2015-07-14

**Authors:** I. Bianchi, G. Bokelmann, K. Shiomi

**Affiliations:** ^1^ Institut für Meteorologie und GeophysikUniversität WienViennaAustria; ^2^ National Research Institute for Earth Science and Disaster PreventionTsukubaJapan

**Keywords:** Anisotropy, Norther Japan, Receiver Function, crustal deformation

## Abstract

Northern Japan is a tectonically active area, with the presence of several volcanoes, and with frequent earthquakes among which the destructive *M*_*w*_ = 8.9–9.0 Tohoku‐oki occurred on 11 March 2011. Tectonic activity leaves an imprint on the crustal structures, on both the upper and the lower layers. To investigate the crust in northern Japan, we construct a receiver function data set using teleseismic events recorded at 58 seismic stations belonging to the Japanese National (Hi‐net) network. We isolate the signals, in the receiver function wavelet, that witness the presence of anisotropic structures at depth, with the aim of mapping the variation of anisotropy across the northern part of the island. This study focuses on the relation among anisotropy detected in the crust, stresses induced by plate convergence across the subduction zone, and the intrinsic characteristics of the rocks. Our results show how a simple velocity model with two anisotropic layers reproduces the observed data at the stations. We observe a negligible or small amount of signal related to anisotropy in the eastern part of the study area (i.e., the outer arc) for both upper and lower crust. Distinct anisotropic features are observed at the stations on the western part of the study area (i.e., the inner arc) for both upper and lower crust. The symmetry axes are mostly E‐W oriented. Deviation from the E‐W orientation is observed close to the volcanic areas, where the higher geothermal gradient might influence the deformation processes.

## Key Points

- Analysis of receiver functions for 58 stations in northern Japan
- Anisotropy is located in both the upper and lower crust
- Anisotropy displays E‐W orientation in the lower crust

## 1 Introduction

Seismic anisotropy is the rock property describing the variation of the speed of seismic waves with the propagation direction at a given point. Causes of anisotropy in the crust are generally explained by the presence of cracks, rock fabrics, or mineral alignment [*Babuška and Cara*, 1991; *Okaya and Christensen*, 2002; *Okaya and McEvilly*, 2003]. Organized cracks play a significant role in the upper crust, while the lower crust is normally more affected by the foliation or lineation of metamorphic rocks [e.g., *Sherrington et al.*, 2004] where anisotropy can reach high values such as 20%. As in many earlier papers, e.g., from *Levin and Park* [1997] to *Schulte‐Pelkum and Mahan* [2014], we assume anisotropy with hexagonal symmetry axes. This can be described either with a unique symmetry axis which is fast(er) or slow(er) than the velocity on the perpendicular plane. For a hexagonally symmetric anisotropic medium, with one unique seismically fast or slow symmetry axis and uniformly slow or fast velocities perpendicular to the axis, five elastic constants are required to fully determine the *P* and *S* wave velocities as a function of propagation direction through the medium. Of these five constants, two are the angles to define the orientation of the symmetry axis and three are the anisotropic parameters, i.e., percent *P* and *S* anisotropies and ellipticity, that is considered as constant (for the parameterizations of hexagonal symmetry anisotropy, see *Sherrington et al.* [2004, and references therein]). Recently, anisotropy within the crust has been demonstrated in regions of active extension and crustal thinning, such as Tibet [*Shapiro et al.*, 2004; *Chen et al.*, 2009] and Western United States [*Moschetti et al.*, 2010], and in areas of high tectonic activity [*Porter et al.*, 2011]. Uniform seismic anisotropy at large scales within the crust might be related to a number of geologically feasible scenarios. Among these are aligned microcracks, developed due to a nonhydrostatic stress field in the shallow crust and alignment of mineral grains in a large body of rock [*Rabbel and Mooney*, 1996]. Cracks are most important in the upper crust, where pressures are low enough to allow them to remain open. The general importance of cracks exclusively at low pressures is supported by experimental studies of rock anisotropy at increasing pressure, which have found that dry cracks close at around 100–200 MPa and no longer contribute to bulk rock anisotropy for greater confining pressure [*Okaya et al.*, 1995; *Barroul and Kern*, 1996; *Weiss et al.*, 1999]. While cracks may be important at shallow depths, numerous studies have found that aligned minerals are the most likely cause of anisotropy in rocks at middle to lower crustal depths [*Rabbel et al.*, 1998; *Weiss et al.*, 1999; *Barroul and Kern*, 1996]. Surprisingly, even though a variety of minerals are common in rocks and most minerals are considerably anisotropic as single crystals [*Babuška and Cara*, 1991], a small number of minerals seem to dominate the bulk anisotropy of most rock types. In particular, micas, such as biotite and muscovite, typically have cleavage planes aligned with a foliation, while amphibole and sillimanite commonly have crystallographic axes aligned with a lineation in strained rocks; these minerals are often the primary cause of bulk rock anisotropy, even if they are not the most abundant minerals in a rock [*Barroul and Kern*, 1996; *Weiss et al.*, 1999; *Mainprice and Nicolas*, 1989]. Anisotropy has been related to the stress field in the upper crust [e.g., *Boness and Zoback*, 2006]; in particular, stress‐aligned fluid‐filled cracks are considered as the cause of seismic anisotropy [*Crampin et al.*, 1986]. For the deep crust there are two other fundamental causes of seismic anisotropy: sequences of finely alternating layered rocks that are effectively anisotropic at large wavelengths even if the rocks' individual layers are isotropic (so‐called layering anisotropy) [see, e.g., *Backus*, 1962] and rock fabrics (lattice‐ and shape‐preferred orientation of minerals) within the structural framework of foliation and lineation that causes anisotropy because most rock‐forming minerals are anisotropic [see, e.g., *Gebrande*, 1982]. A laminated lower crust composed of individually isotropic layers would only be weakly anisotropic or even isotropic [cf. *Rabbel and Lüschen*, 1996]. Most probably, a possible lower crust anisotropy would be due to rock fabrics, namely, the lattice‐preferred orientation of highly anisotropic minerals such as biotite, hornblende, etc. [e.g., *Mainprice and Nicolas*, 1989], and the textural alignment of minerals in metamorphic rocks is mainly caused by ductile flow. Common to all these causes is that the geological process is an ordering process characterized by a directional dependence itself, such as sedimentation, tectonic stress, or ductile deformation.

The motivation of this work is to study anisotropy in the crust beneath northern Japan (NJ) and its relation with present‐day tectonics. Here we observe some parallelism between the present‐day stress field and anisotropy orientation. The first known indicators of stress field are focal mechanisms of earthquakes that took place in crustal media; although they can be biased by the presence of major faults, the orientation of which may deviate from the background field. *Yoshida et al.* [2012] show the stress field for crustal earthquakes. It is E‐W oriented before the occurrence of the Tohoku‐oki earthquake. First, *Kaneshima* [1990] and later, *Huang et al.* [2011a, 2011b] apply the shear wave splitting method to NJ to obtain an estimate of the anisotropy orientation. The retrieved anisotropy orientations are roughly E‐W for the older study, but it is different in the more recent application. In the first study, the splitting is assumed generated in the shallow crust (first 15 km), as the layer that most affects the recorded signal, and the anisotropy in the lower crust is treated as relatively weak. In the latter work, the splitting parameters show a thick anisotropic layer at the base of the crust. In these works, the spatial distribution (in both depth and lateral) of the measurements depends exclusively on the earthquakes location. Our study shows that upper and lower crustal anisotropic characteristics might differ in their orientation and might be affected by the presence of major structural elements such as faults, dykes, and magmatic chambers. To illuminate the crustal anisotropic characteristics in NJ, we use a method that is able to robustly determine the effect of anisotropic material at the desired scale in the crust: the interpretation of teleseismic receiver functions (RFs) including both radial (R) and transverse (T) components. The effects of anisotropy on the RF data set were illustrated in more than one theoretical study [e.g., *Eckhardt and Rabbel*, 2011; *Nagaya et al.*, 2008; *Levin and Park*, 1998], showing the strong back azimuthal dependence of RF on the 3‐D characteristics of the media traversed by the rays. This technique was applied in several places around the world with the aim of creating realistic velocity models beneath single stations or wide areas [e.g., *Savage*, 1998, 1999; *Ozacar and Zandt*, 2009; *Bianchi et al.*, 2008; *Liu and Niu*, 2012; *Porter et al.*, 2011]. The use of teleseismic RF brings some advantages with respect to the previous cited techniques, since they are not dependent on the local earthquakes distribution. At depth, the teleseismic wave samples the whole crust, and in NJ the dense distribution of seismic stations allows a large number of observations. This technique has been applied in this area by *Wirth and Long* [2012] to focus on the mantle wedge and slab‐related anisotropy; we focus here on the crust with the aim of constraining the depth of the anisotropic layers and the orientation of anisotropy symmetry axes. In a recent paper, *Watanabe and Oda* [2014] also estimate the anisotropic structure of the upper crust, lower crust, and mantle wedge by stripping analysis of the *P* *s* phases and *S* waves; their results are consistent with those of *Nakajima and Hasegawa* [2004] from shear wave splitting in the area. Both studies find E‐W oriented fast anisotropy axes in the western side of the study area and N‐S oriented axes in the eastern side of the area.

## 2 Tectonic Setting

The principal tectonic element of northern Japan is the subduction of the Pacific Plate beneath the arc, with a rate of ≈9 cm/yr [*DeMets et al.*, 1994]. The geologic structure of northern Japan is the result of several phases of tectonic activity, which can be summarized in four stages [*Sato and Amano*, 1991].

1. A prerifting stage (before 25 Ma).
2. An extensional stage (25–13 Ma), where half‐grabens are formed under an extensional regime related to the rifting of the Sea of Japan and the basins are filled with volcanic products. Crustal extension within the arc occurred in a later stage (16–13 Ma) with subsidence causing marine incursion [*Sato*, 1994].
3. A neutral stage (13–3.5 Ma) with about 10 Myr of tectonic quiescence.
4. A compressive stage (3.5 Ma to present) with crustal shortening perpendicular to the arc. The principal morphotectonic feature of the arc is the N‐S uplifting volcanic range called Ou Backbone Range, which defines the volcanic front of the arc.

**Figure 1:**
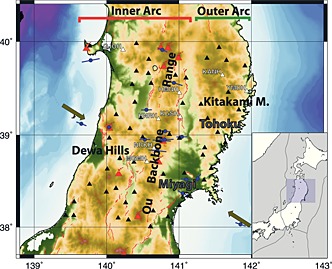
Map of Tohoku region. The three major tectonic regions are highlighted: The Kitakami Mountains in the east, the Ou Backbone Range in the center, and the Dewa Hills in the west. Black triangles represent the seismic station locations, white triangles for stations whose data are shown in this paper. Red symbols for active volcanoes and for active faults [*Nakata and Imaizumi*, 2002]. Green arrows for plate motion (from University NAVSTAR Consortium (UNAVCO)—EU versus EA and EA versus EU). Blue bars for orientation of maximum horizontal compressive stress orientation (from the World stress map project [*Heidbach et al.*, 2008]).

Active volcanoes occur sporadically west of the front. The Ou Backbone Range is one of a series of antiforms which accommodate the compression of the arc, being an active pop‐up structure, bounded by opposite facing reverse faults [*Sato and Amano*, 1991]. The tectonic setting is the result of the Miocene (25–13 Ma) rift‐induced extension which formed deep depositional basins that are N‐S oriented. Their original bounding faults have been reactivated as inverse faults by the later (still ongoing) compressional regime. The Japan Trench subduction zone has hosted nine events of magnitude 7 or greater since 1973. A number of strong reverse‐slip earthquakes have occurred over the past years in the upper crust of NE Honshu on both sides of the volcanic front; in several cases the ruptures are classed as compressional inversion earthquakes [*Sibson*, 2009, and references therein], happening on the reactivated basin border faults. These faults follow the general N‐S trend of the extensive‐regime‐generated structures (e.g., Senya fault [*Sato et al.*, 2002]), while in the Miyagi region, the rupture planes are NW oriented (e.g., Northern Miyagi [*Okada et al.*, 2008]). The Japanese arc is divided into outer arc and inner arc (Figure 1). In the Tohoku region the outer arc is represented by the Kitakami Massif. This massif is composed of Paleozoic to Mesozoic sedimentary rocks, intruded by granites [e.g., *Minato et al.*, 1965]; it is a stable arc block. The outer arc is a nonvolcanic region, with gentle slopes resulting from dissection of long duration. This mountain range has never been under the sea and has not been subject of intensive crustal movement during the Cenozoic. The inner arc is divided in two narrow uplifted zones: (1) The Ou Backbone Range, active since early Miocene with more than 2000 m thick felsic volcanic pile and now characterized by large caldera structures. The Ou Range, about 500 km long, runs north‐south and is the longest range in Japan. The Ou Range began to uplift in the late Miocene and the uplift rate increased in the Quaternary. (2) The Dewa Hills, made up of submarine volcanic and argillaceous sediments (about 2000 m thick). These are young fold mountains, which started to upheave in the Late Pliocene and are separated into several mountain ranges by antecedent valleys. In the inner arc region, short‐wavelength folding and faulting are dominant, forming mountains and basins smaller than those in the outer arc region. This region is being compressed east‐west [*Hasegawa et al.*, 2009; *Loveless and Meade*, 2010] and is composed of formations (mainly of the Neogene) younger than those of the outer arc region; moreover, the high ground temperatures, due to the rising magma, facilitate the deformation of the layers.

## 3 Data and Methods

### 3.1 RF Computation

Data used for this study have been collected at 58 stations (Figure 1) belonging to the National Institute for Earth Science and Disaster Prevention Hi‐net. The network is uniformly distributed on the Japanese Island with an interstation spacing of 20–30 km [*Obara et al.*, 2005]. The stations used in this study are located between 38^∘^ and 40^∘^N latitude. Teleseismic records collected between January 2009 and December 2010 have been first selected based on their magnitude (*M*_*w*_ ≥ 5.7) and epicentral distance (*Δ*, 30 > *Δ* <100). Later the selected teleseisms have been picked and again selected through an automatic procedure, according to their short‐term average/long‐term average ratio, through which we exclude seismic waveforms with a low S/N ratio, discarding the waveforms with a nonclear arrival of the *P* direct phase. A window of 120 s around the *P* arrival time was selected, the traces downsampled to 25 samples per second and the seismograms rotated to the RTZ reference system, where the radial (R) is computed along the great‐circle path between the epicenter and the station, positive away from the source, and the transverse (T) direction is calculated 90^∘^ clockwise from R. RFs were computed following the multitaper approach developed by *Park and Levin* [2000] with two low‐pass filters; at 2 Hz to focus on the upper crust and 1 Hz to focus on the lower crust. We obtained a final data set of about 15,000 three‐component records, with a minimum of 113 and a maximum of 403 records for single station. RFs are plotted in back azimuth bins of 10^∘^. Each bin contains all the incoming rays with back azimuths ±5^∘^ of the bin value. The back azimuthal coverage for each station is almost complete.

### 3.2 Data Set Analysis

**Figure 2:**
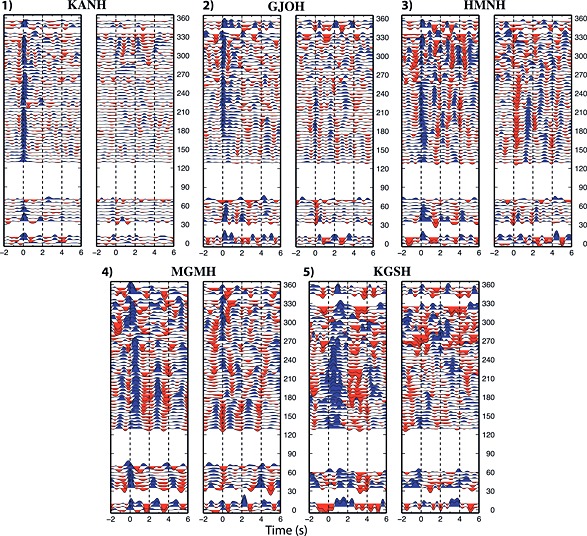
Examples of data set for the five quality classes of receiver functions: 1 for the best quality and 5 for the stations excluded from this study. Radial and transverse components of the receiver functions are shown on the left and right, respectively. Blue and red pulses correspond to positive and negative velocity jumps, respectively. On the vertical axis, the back azimuth angle (clockwise from north). On the horizontal axis, time in seconds after the arrival of the *P* direct phase.

The 58 stations have been classified according to five quality levels (see Figure 2). Quality 1 for the best stations, to quality 5 for the discarded data sets. The teleseism selection shows the same back azimuthal distribution for each station in the pool. According to this, the data set classification is not biased by the azimuthal coverage of the events for the single station. In Table 1 the stations have been grouped according to their geographical location (inner or outer arc) and one quality value has been assigned. Quality (Q, hereinafter) 1 has been assigned to stations showing low amplitudes at negative delay times, showing the *P* direct phase picked at 0 arrival time, and low signal on the T component. Data from quality 1 stations are easier to interpret since the signal on T component is missing; this means that the structure below these stations is lacking significant 3‐D features such as dipping layers or anisotropy and, therefore, there are less parameters to constrain in the velocity model. In some cases, as in the example in Figure 2, the Moho phase is visible (at 5 s) but not prominent. Q2 has been assigned to stations showing low amplitudes at negative delay times, showing the *P* direct phase picked at 0 arrival time, visible Moho on R component and visible signal on the T component. Q3 has been assigned to stations showing higher amplitudes at negative delay times, showing the *P* direct phase picked at 0 arrival time, visible Moho on R component and visible signal on the T component. Q4 has been assigned to stations showing higher amplitudes at negative delay times, showing a *P* direct phase shifted from 0 arrival time, visible Moho on R component, and high signal on the T component. Q5 has been assigned to stations showing higher amplitudes at negative delay times, showing a *P* direct phase shifted from 0 arrival time and high signal on the T component, here the continuity of *P* *s* phases is hard to follow along the back azimuthal sweep. To the stations located on the outer arc the quality 1 has been assigned, with the exception of the stations SNDH (Sendai). The reason for this homogeneous quality class is the crustal structure beneath the outer arc, consisting of a stable granitic crust and characterized by the absence of shallow sedimentary layers. RFs obtained for stations located otherwise are influenced by the crustal structure; shallow strong acoustic impedance contrasts cause some ringing which affects the whole signal, or the presence of dykes and abrupt changes in the subsurface structure, as is known in volcanic areas, might cause signals with unexpected large amplitudes, or features in the data set in which continuity is interrupted along the back azimuthal sweep. The method used to estimate the RF (multitaper spectral correlation by *Park and Levin* [2000]) provides an estimate of RF uncertainty in the frequency domain, enabling RF from different seismic events to be combined in a weighted‐average RF estimate, rather than an unweighted stack. This method appears to be more resistant to incoherent signal‐generated noise, which can seriously contaminate RF estimates and might arise from the stacking of events arriving from different epicentral distances. In Figure 2, examples of stations for each quality data set computed with a low‐frequency filter of 2 Hz are shown.

**Table 1:** Seismic Stations Information

				Location		
Station				0 = Outer Arc	Upper Crust Anisotropy	Lower Crust Anisotropy
Name	Longitude	Latitude	Quality	1 = Inner Arc	Trend (^∘^ From N)	Trend (^∘^ From N)
FSWH	141.3547	38.8625	1	0	288	71
IWZH	141.6556	39.7992	1	0	314	119
KAKH	141.3456	38.5129	1	0	245	63
KANH	141.6013	39.6414	1	0	301	97
KASH	141.6811	39.4601	1	0	99	69
KKWH	141.6412	38.9178	1	0	230	62
KMIH	141.8269	39.2712	1	0	248	41
KZMH	141.5528	39.9369	1	0	108	129
RZTH	141.5356	39.0278	1	0	126	54
SMTH	141.3944	39.1781	1	0	117	66
SNDH	141.0003	38.237	2	0	298	90
SZGH	141.4463	38.6386	1	0	292	117
TAJH	141.0744	38.5878	1	0	295	93
TMYH	141.3861	39.8222	1	0	103	80
TOUH	141.305	39.3311	1	0	270	40
TOWH	141.3289	38.7831	1	0	225	70
TROH	141.9123	39.7407	1	0	87	63
YMDH	141.9372	39.4705	1	0	256	87
AHIH	139.5519	38.2769	4	1	284	57
ANIH	140.4107	39.9791	3	1	281	110
ARKH	139.4322	38.1297	5	1	299	123
ASAH	139.7639	38.4675	1	1	78	116
CHKH	140.3219	39.0689	1	1	93	53
FGTH	140.3778	38.7078	1	1	297	71
GJOH	140.2256	39.9128	2	1	258	107
HMNH	141.0036	39.4531	3	1	316	46
HMSH	141.0508	39.3406	4	1	260	62
HNRH	140.7164	39.1711	1	1	230	58
ICEH	141.0047	38.9661	2	1	127	90
IWNH	140.8475	38.1103	5	1	330	99
KGSH	141.0153	39.195	5	1	310	94
KMYH	140.3011	38.0783	1	1	248	112
KWAH	140.411	39.6857	1	1	138	81
KWSH	140.6439	38.1772	3	1	109	143
MGMH	140.5578	38.7103	4	1	153	80
NKEH	140.1283	38.4228	2	1	221	79
NKWH	139.995	38.3831	4	1	305	81
NRKH	140.6547	38.8558	1	1	33	102
NSBH	140.3515	39.5422	5	1	265	64
NSEH	140.615	39.5547	5	1	274	106
NSNH	140.5825	39.8119	4	1	250	100
NSSH	140.5756	39.6606	1	1	318	73
NYOH	140.1586	38.1006	1	1	239	71
OGAH	139.7657	39.9527	4	1	283	90
OGCH	140.4986	38.9772	3	1	304	59
ONDH	140.7839	38.5764	4	1	120	102
SISH	140.6061	38.0061	2	1	68	128
SKWH	139.5461	38.1283	5	1	101	98
SZKH	140.95	39.6408	2	1	298	72
TCKH	140.0089	38.6328	4	1	244	69
TDOH	140.3839	38.3811	4	1	100	83
TZWH	140.1814	38.7469	1	1	246	59
YGTH	140.2617	38.2664	5	1	99	90
YHBH	141.0964	39.6119	2	1	293	67
YJMH	140.1317	39.2194	3	1	283	95
YUZH	140.4744	39.1885	4	1	289	80
YWTH	140.0367	38.9672	2	1		46

### 3.3 Data Processing for RF Harmonic Decomposition

**Figure 3:**
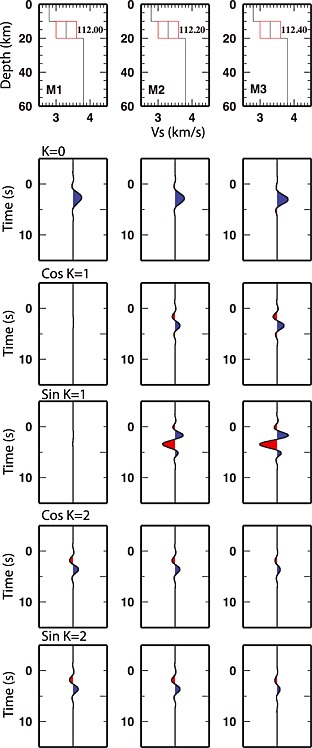
Example of harmonic components of synthetic RF data sets with the reference velocity model. (left column): Velocity model (top) and the five components of the harmonics expansion for a layer with horizontal anisotropy axis, N112 oriented. (middle column): Velocity model (top) and the five components of the harmonics expansion for a layer with 20^∘^ dipping anisotropy axis, N112 oriented. (right column): Velocity model (top) and the five components of the harmonics expansion for a layer with 40^∘^ dipping anisotropy axis, N112 oriented (*θ* = azimuth).

The method of analysis is based on the extraction of the back azimuthal harmonics of an RF data set as a function of the incoming *P* wavefield direction [*Girardin and Farra*, 1998; *Farra and Vinnik*, 2000]. At every time step an ensemble of RF can be expressed as a scaled sum of cos *k* *φ* and sin k*φ*, where *k* is the harmonic degree (0, 1, and 2) and *φ* is the back azimuth. The RF image is composed of five gathers: (1) a stack of the R‐RF for each ensemble (for *k* = 0); (2) the first‐order (*k* = 1) cosine component of the harmonic analysis (in which maximum amplitude occurs for north or south dipping interfaces or north or south trending anisotropy axes); (3) the first‐order (*k* = 1) sine component of the harmonic analysis (in which maximum amplitude occurs for east or west dipping interfaces or east or west trending anisotropy axes) [see *Bianchi et al.*, 2010; *Bianchi and Bokelmann*, 2014, for details]; (4) the second‐order (*k* = 2) cosine in which maximum amplitude occurs for both N‐S or E‐W trending anisotropy with a horizontal symmetry axis; and (5) the second‐order (*k* = 2) sine component, in which maximum amplitude occurs for anisotropy with a horizontal symmetry axis, trending at 45^∘^ (i.e., N45^∘^, N135^∘^, N225^∘^, and N315^∘^). The E‐W and N‐S distinction for *k* = 1 and the N45^∘^, N135^∘^, N225^∘^, and N315^∘^ component separation for *k* = 2 simply arise from the sine and cosine terms of the phase. Figure 3 shows the different amplitudes for *k* = 1 and *k* = 2 according to the plunge of the anisotropic symmetry axis. Common features of the velocity models in Figure 3 (summarized in Table 2) are the isotropic velocity jumps at 10 and 20 km depth. The differences among the models in Table 2 are due to the plunge of the anisotropy symmetry axis; being horizontal for model *M*1, plunging of 20^∘^ toward ESE in model *M*2, and plunging of 40^∘^ toward ESE in model *M*3. Trend of the anisotropic symmetry axis for the three models is N112^∘^. Figure 3 shows clearly the increasing amplitudes on the first harmonic components (*k* = 1) with the increasing plunge of the symmetry axis. However, the second component of the harmonics (*k* = 2) shows decreasing amplitudes for increasing plunge of the symmetry axis. A slightly plunging anisotropic axis, as, e.g., 20^∘^ from horizontal, causes a 360^∘^ periodicity in the RF data set, which is reflected in the *k* = 1 term of the harmonic analysis. Thus, the interpretation of the *k* = 1 gives by itself enough information to determine the orientation of the symmetry axis of anisotropy and normally shows larger amplitudes than the *k* = 2 term. The interpretation of the *k* = 2 term is considered, when it shows larger amplitudes than the terms *k* = 1. This happens for horizontal anisotropy axis, for which RF data set displays a 180^∘^ periodicity in the back azimuthal sweep. The completeness of data sets allows a stable harmonic analysis. The method is not subject to the presence of the noise as each wiggle is a weighted sum of all the RF (R and T), so the noise is attenuated through the weighted sum.

**Table 2:** Velocity Model Used for Calculating Synthetics Waveforms in Figure 3

Thickness (km)	Vp (km/s)	Vs (km/s)	Anisotropy Trend	Anisotropy Plunge
10	4.8	2.8	‐	‐
10	5.7	3.3	N112^∘^	(M1) 0^∘^, (M2) 20^∘^, (M3) 40^∘^
40	6.7	3.8	‐	‐

### 3.4 Modeling

We solve the RF inverse problem for one example station in order to get a velocity model comprehensive of anisotropic characteristics of the media and to assess a confidence interval. The inverse problem was solved by the application of the Neighborhood Algorithm (NA). The algorithm samples iteratively the good data‐fitting region of a given parameter space and extracts information on the 3‐D crustal structure [*Sambridge*, 1999]. Following the original implementation of the NA, we initially generated 1000 samples from the parameter space. From the neighborhood of the five best fit models, 50 new samples are iteratively resampled. After 100 iterations we obtained an ensemble of 6000 models. The method outputs one best fit model for each iteration and extracts the global best fit model. The output values of the 6000 best fit models describe a Gaussian distribution and are used to define the mean and standard deviation values to assess a confidence interval on the anisotropy orientation and percentage. Synthetic receiver functions for an anisotropic medium were calculated using RAYSUM [*Frederiksen and Bostock*, 2000], a ray‐based technique that calculates the amplitude and traveltimes for different phases, which are then combined to create a synthetic seismogram. In this study we use a slightly modified version of the code by *Frederiksen et al.* [2003] to correspond to the parametrization of *Levin and Park* [1998] for the case where the anisotropy is assumed to be purely ellipsoidal (same parameterization as in *Sherrington et al.* [2004]). The technique is limited to the plane wave assumption, i.e., teleseismic waves. We did not calculate multiples in our modeling because the multiples from the lower crust arrive much later than the time window in which we wanted to focus (as in *Sherrington et al.* [2004]). RAYSUM allows including the effect of dipping layers. Dipping layers can trade off against anisotropy, as observed by comparing models above the Pacific subducting slab by *Savage* [1998] and *Savage et al.* [2007]; we avoid modeling such features in order to keep our focus on the effects due to anisotropy. The effects of dipping boundaries are usually extremely strong in the very shallow upper crust (upper few kilometers below the station, where the sedimentary layers get in contact with the basement); in this study we do not want to solve for the very upper shallow layers but rather provide a simple model including two layers of anisotropy.

### 3.5 Parameter Space and Number of Anisotropic Layers

The parameter space (summarized in Table 3) has been studied to focus on the anisotropic characteristics of the media. Velocities of the layers and thicknesses are defined to allow small changes to the model; the reference velocity model was inspired by previously published works of *Xia et al.* [2007] and *Zhao et al.* [1992]. We interpret the signals as being due to anisotropy and not to dipping interfaces due to the occurrence of coupled phases, i.e., two consecutive pulses of opposite polarity; while an inclined interface would result in just one pulse [see *Bianchi et al.*, 2010]. Values related to anisotropic characteristics of the media, such as trend and plunge of the axis, and percent of anisotropy, are set to explore the whole range of possibilities. The number of anisotropic layers is set to two with fast anisotropy axes; the layers are located one in the upper crust and one in the lower crust as inferred from the visual inspection of the harmonically expanded signal from the entire database of RF, calculated for the 58 stations. We explore only the possibility of anisotropy with plunging symmetry axis, since a horizontal symmetry axis would generate signal on the *k* = 2 components of the harmonic decomposition. Anisotropy with a vertical axis would not show in the RF signal, since rays coming from different back azimuths would not show differential arrival times.

**Table 3:** Maximum and Minimum Velocity Models Defining the Parameter Space for the NA Search, Together With the Best Fit Model

Thickness (km)	Vs (km/s)	Vp/Vs	Anisotropy %*P* and *S*	Trend	Plunge
*Model Minimum/Maximum*					
6/10	3.2/3.5	1.72			
6/15	3.2/3.5	1.72	2.0/8.0	90/270	10/50
2/10	3.2/3.7	1.76			
10/20	3.5/3.8	1.76	4.0/8.0	0/180	10/50
Half‐space	4.1	1.81			
*Best Fit Model*					
7.5	3.2	1.72			
9	3.4	1.72	4.2	143	48
3	3.6	1.76			
13	3.6	1.76	5.3	118	18
Half‐space	4.1	1.81			

## 4 Results

### 4.1 Anisotropy Search

**Figure 4:**
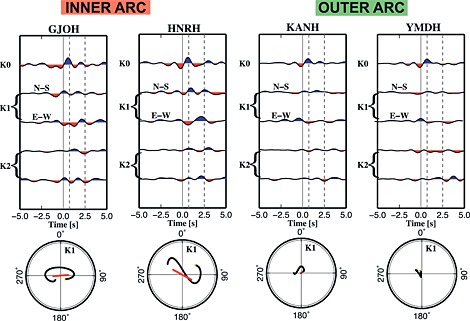
Examples of harmonic decomposition of RF data set for stations located on the inner and outer arc. Particle motion is calculated for delay times between 0.7 and 2.5 s of the *k* = 1 terms (5 to 20 km depth). The particle motion is then interpreted with its linear fit (red arrow). Inner arc: Large amplitudes are detected, caused by anisotropy in the upper crust. Outer arc: Weak signal is detected, corresponding to weak or no anisotropy for these stations.

Our goal is to determine trend and plunge of the anisotropic symmetry axes at each station and then show the variation of anisotropy along and across the island for the upper and lower crust. Each station has enough data to give stable results after the application of the harmonic expansion. The two terms of the first‐order harmonic (*k* = 1) are used to constrain the orientation of this anisotropic axis. According to the construction of the harmonic components, the first term is maximized when a N‐S oriented structure is present, while the second one is maximized for E‐W oriented structures. Following this rule, the combination of signal inside a limited time window shows the azimuthal orientation of the anisotropy axis, and the polarity of the pulses instead indicates the trend of the symmetry axis; where north and east trending axes generate a first pulse of positive polarity (blue) and south and west trending axes generate a first pulse of negative polarity (red). The linear fit of the particle motion of the two *k* = 1 terms (in a defined time window) indicates the orientation (i.e., the two opposite plunge are possible, as in Figure 4) of the anisotropic axis, while the polarity of the pulses reveals the plunge of the axis. The amplitude is dependent on both the plunge of anisotropy symmetry axis and the anisotropy strength. There is a tradeoff between the two parameters. Individual uncertainties of these parameters, plunge, and anisotropy strength are therefore hard to specify and make the interpretation not straightforward. In our modeling, a 2% *P* and *S* anisotropy corresponds to a 50^∘^ plunge of the anisotropic axis, while an 8% of *P* and *S* anisotropy corresponds to a 10^∘^ plunge of the anisotropic axis. We used here the pulse amplitude as a proxy to indicate the strength of anisotropy. Therefore, the most robust result of our work is the orientation of the anisotropy axes, and we will discuss this only.

**Figure 5:**
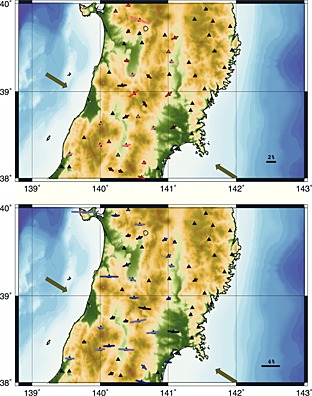
Map displaying the particle motion calculated on the *k* = 1 term of the harmonic analysis for single station. (top) Red for the upper crust and (bottom) blue for the lower crust. Darker colors for higher‐quality classes. Length is proportional to percent anisotropy and orientation is the orientation of the fast axis.

For all stations, the particle motion from which we retrieve the axis orientation has been calculated in two characteristic time windows: 0.6–2 s (representing the upper crust) and between 2.5 and 5 s (representing the lower crust) for RF calculated at two different corner frequencies, as 2 Hz for the upper crust and 1 Hz for the lower crust. The Moho in northern Tohoku is 35 to 40 km deep [*Katsumata*, 2010]; therefore, considering the signal arriving by 5 s, we do not include and interpret features generated in the mantle. Results are displayed on a map separately for the upper and lower crust (Figure 5). For some stations the time window for the lower crust was shifted according to the arrival time of the major intracrustal interface (probably the Conrad) pulse, and the Moho pulse arrival time: ASAH 3–5 s, OGCH 4–6 s, HNRH 3–5 s, and NRKH 2.4–4.5 s. Search results for the entire pool of stations are expressed in Table 1 and shown in Figure 5.

### 4.2 Confidence Interval

**Figure 6:**
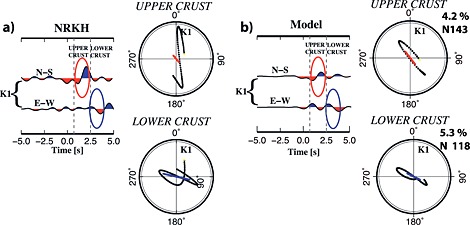
(a) The *k* = 1 harmonic components calculated for the station NRKH and particle motion defined for the upper crust and lower crust. (b) The *k* = 1 harmonic components from the synthetic data set obtained after the modeling and its relative particle motion for upper and lower crust; values of anisotropy trend and % are shown too. Ovals highlight the pulses fitted by the anisotropic parameters of the two layers (red for the upper crust and blue for the lower crust).

**Figure 7:**
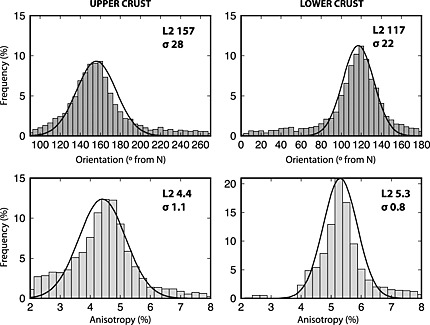
Count of single‐iteration best fit values for anisotropy at stations NRKH: (top row) fast direction for (left column) upper and (right column) lower crust and (bottom row) anisotropy percentage. Histograms are fit with the Gaussians, the mean (*L*2) and standard deviation (*σ*) of which are reported in each diagram.

A confidence interval is defined for two parameters: trend of anisotropy and anisotropy percent for the upper and for the lower crust. The modeling procedure was applied to the station NRKH; observed and synthetic waveforms are in Figure 6. The results of the modeling show how the signal included in the time interval related to the upper crust is poorly fit by the data; in the upper crust, indeed, the structures are more heterogeneous and the signal due to lateral variations or steeply dipping interfaces might affect the signal related to anisotropic layers; we should therefore consider a high uncertainty on the anisotropy trend in the upper crust. The anisotropic signal in the lower crust instead is well fit. Anisotropy trend and percent for upper and lower crust obtained for the 6000 models are shown in Figure 7 and display a Gaussian distribution; the standard deviation (*σ*) defines an estimate of the confidence interval. Mean (*L*2) and *σ* are reported in Figure 7 for each Gaussian.

### 4.3 Upper Crust

**Figure 8:**
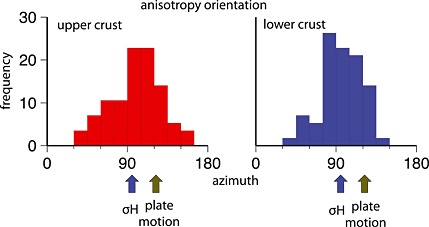
Frequency histogram showing the distribution of symmetry axes orientation for upper (red bars) and lower (blue bars) crust. The blue arrow shows the average orientation of *σ*_*H*_ (World stress map project [*Heidbach et al.*, 2008]); the green arrow shows the direction of plate motion (from UNAVCO EU versus EA and EA versus EU).

In the upper crust we find a strong difference between stations located on the outer arc and those on the inner arc. The length of the arrows in the maps of Figure 5 corresponds to the shear wave anisotropy; it is scaled according to the results of the synthetic data set obtained after the modeling. The different colors of the arrows are related to the quality class of the station (darker color for best quality). The stations on the outer arc display negligible amplitude of the anisotropic signal, while the stations located on the inner arc, especially on the Ou Backbone Range, display a larger amount or steeper dips of anisotropy. The fast axes of anisotropy are mostly ENE‐WSW oriented, and five of them are ESE‐WNW oriented (see Figures 5 and 8).

### 4.4 Lower Crust

In the lower crust we observe the same separation between the outer and inner arc, a negligible amount of anisotropy is found at the stations located on the outer arc and a larger amount at the station on the inner arc. In the lower crust the orientation of the anisotropic fast axes is distinctly E‐W (see Figure 8). The E‐W orientation of these axes and the strength of anisotropy are both more marked for the stations located south of 39^∘^N latitude.

## 5 Discussion

In this study we observe two regions of anisotropy in the crust. The first region of anisotropy is within the upper crust, at depths between about 5 and 15 km that are probably too deep for significant crack systems to remain open. Anisotropy at these depths is thus probably due to alignment of mineral grains, yet this part of the crust is not likely at appropriate temperature and pressure conditions to have experienced recent ductile deformation and/or recent widespread development of metamorphic fabrics, and the anisotropy that we detect might be inherited from past events. Although the past motion of the Pacific Plate is still unclear, if the Hawaiian‐Emperor chain has been constructed by a stationary hot spot, the Pacific Plate moved to NNW until 40 Ma ago. Though this hypothesis has been rejected based on the paleomagnetic data [*Tarduno et al.*, 2003], we may say that the plate motion has been almost stable during the last 40 Ma, while the opening of the Sea of Japan began about 30 Ma. During this opening procedure, northern Japan rotated anticlockwise. Before the opening (as shown in *Kimura et al.* [2005]), the plate motion direction was almost perpendicular to the present volcanic front (Ou Range). This means that the fast axis orientation estimated in this study corresponds well to the past plate motion direction. The vicinity of the volcanic edifices might anyway change the local conditions and therefore induce a ductile deformation; such a phenomena should show local patterns. The second depth zone of anisotropy is in the middle to lower crust, and anisotropy at these depths may be a result of recent or ongoing ductile deformation, metamorphism, or crustal flow.

### 5.1 Comparison With Previous Results and Validity of Anisotropy Orientation

#### 5.1.1 Upper Crust

Anisotropy in the upper crust is stronger beneath the Ou Backbone Range, which represents the highest crest of the area and includes several volcanoes. The presence of volcanism could strongly affect our results on orientation and amount of anisotropy beneath the stations and would also explain the variable orientation of the symmetry axes. Volcanic areas present, indeed, abrupt lateral variation with the presence of dykes and of magma chambers; moreover, the area is populated by faults bounding the sedimentary basins; all these structural features might generate phases that interfere with the phases due to the anisotropy in the upper crust; we therefore rely less in the anisotropy orientations obtained for the upper crust with respect to those obtained for the lower crust. The anisotropy reflects the different structures in the shallow crust, being complex for the Dewa Hills and Ou Backbone Range, and missing at the Kitakami Mountains. Information from previous studies confirms the lack of parallelism among the anisotropic axes in the upper crust [*Huang et al.*, 2011a]; weak anisotropy has been inferred in the upper crust by *Nakajima and Hasegawa* [2004] and by *Watanabe and Oda* [2014]. The orientation of the unique symmetry axis is the most robust parameter that we infer, either fast or slow; previous information and geological and geophysical constraints might help to decrease the nonuniqueness in modeling. An E‐W oriented shear might develop a lineation that dips to the east or to the west according to the nature of the symmetry axis (fast or slow). An alternative explanation could be related to E‐W shear with development of N‐S striking dipping foliation planes.

#### 5.1.2 Lower Crust

Anisotropy is present in the lower crust, displaying an average consistent E‐W orientation. The lower crustal deformation has been thought to be ductile because of the low seismic activity. Anisotropy was previously inferred in the lower crust by *S* wave splitting of the low‐frequency earthquakes near the Moho [*Huang et al.*, 2011a]. The lateral variation clarifies whether anisotropy is a significant feature in the lower crust or not. *Ishise and Oda* [2005] inferred *P* wave anisotropy at different depths beneath northern Japan. A comparison with this work could be speculative, since they create images at determined depth, while we focus at the dominant signals in the data set. *Watanabe and Oda* [2014] infer the splitting parameters of the Moho *P* *s* phase from RF after correction from the upper crustal effects and find E‐W orientation of anisotropy in the western part of the study area and N‐S orientation of anisotropy for the eastern part of the study area. We find in the outer arc that the magnitude of anisotropy is small, meaning a scarce orientation of minerals in this part of the crust, which may reflect a small or absent deformation; alternatively, highly deformed and oriented minerals may not generate anisotropy, depending on composition. Our results for the stations located in the outer arc differ from what were previously obtained by *Watanabe and Oda* [2014]. In the inner arc the lower crust shows strong anisotropic characteristics, with the orientation of the fast anisotropy axes predominantly E‐W. The lower crust is thought to be ductile because seismic activity is significantly low; therefore, the lower crust undergoes plastic deformation in the E‐W direction that might be related to the westward motion of the Pacific slab. This might be enhanced by low Vs due to fluids present in the inner arc (shown in *Wang et al.* [2012]), which occur within the areas of stronger anisotropy and E‐W orientation of the axes detected by our study. The earthquake distribution at depth clearly outlines the difference between outer arc and inner arc. In the outer arc, deep crustal earthquakes occur, while this is not the case in the inner arc, where no earthquakes occur [*Ishise and Oda*, 2005]. This difference outlines the behavior of the two portions of the Japanese arc, the outer deforming brittlely and the inner deforming plastically. According to *Huang et al.* [2011a], in the lower crust the orientation of anisotropy is E‐W where the volcanic activity is less intense, and in this study we confirm this statement showing the loss of parallelism of the axes at the volcanic group located south of 40^∘^N. To the south of 39^∘^N most of the orientations are at 30^∘^–40^∘^ from the direction of convergence of the Pacific Plate against the Eurasian Plate. The average anisotropy intensity of the non‐EW oriented axes is lower than the intensity of the E‐W oriented anisotropy axes. On the overall comparison (Figure 8) fast orientation agree better with the maximum horizontal compressive stress orientation, *σ*_*H*_, rather than the present‐day relative motion between the Pacific and the Eurasian Plates. This is an interesting observation, especially for the lower crust, which might be possibly affected by the intraplate deformation.

The *σ*_*H*_ values in northern Japan result from earthquakes in the upper crustal levels [*Heidbach et al.*, 2008]. We have noticed the parallelism between the orientation of anisotropy in the lower crust and the *σ*_*H*_ orientation. One of the hypotheses is that the *σ*_*H*_ is uniformly oriented with depth.

Moreover, the northern Japanese slab has NS strike, and the E‐W orientation of anisotropy in the lower crust might be related to the subduction of the Pacific Plate [*Hasegawa et al.*, 2009; *Nakajima and Hasegawa*, 2004; *Watanabe and Oda*, 2014]. The interseismic GPS velocity field in *Loveless and Meade* [2010] shows E‐W oriented velocities in the area, with a higher magnitude at the eastern coast and decreasing toward the west. The velocity directions twist toward NW in the northern part of the area. The velocity field, as described, reflects the orientation of the anisotropy axes here detected but with a reversed magnitude (i.e., where the anisotropy is stronger, the GPS velocities are lower, and vice versa). This might lead to the conclusion that the NE Japanese crust responds differently to deformation across the island. It acts as a rigid block in the eastern part (i.e., Kitakami Mountains), where it undergoes small or no deformation (visible as small or no anisotropy) but moving uniformly toward the west as a block (high GPS velocities). The crust deforms plastically in the western part (i.e., Ou Backbone Range and Dewa Hills) where the EW oriented shortening is muffled by the internal deformation of the rocks (i.e., high anisotropy percent).

## 6 Conclusions

From the analysis of a receiver functions data set collected at 58 stations in northern Japan, we are able to detect the presence of anisotropy in the crust. We divided the signal for selected arrival times, corresponding to the upper and to the lower crust. Through the application of the harmonic analysis of the data set, we determine the orientation of anisotropy and create maps for the upper and lower crust. We find a strong difference between the signals recorded at stations located on top of the outer arc and on top of the inner arc. Stations located on the rigid cold outer arc show no anisotropy both for the upper and for the lower crust, while stations on the inner arc show large values of anisotropy for both upper and lower crust. In the upper crust the abrupt variations of the shallow structures, in particular, in the inner arc, where the volcanic structures are developed, do not allow the unique determination of anisotropy orientation. In the lower crust we observe homogeneous E‐W anisotropy orientation. This observation confirms results from previous studies and shows a relation with the westward motion of the Pacific slab.

## References

[jgrb51169-bib-0001] Babuška, V. , and M. Cara (1991), Seismic Anisotropy in the Earth, Kluwer Acad., Boston, Mass.

[jgrb51169-bib-0002] Backus, G. (1962), Long‐wave elastic anisotropy produced by horizontal layering, J. Geophys. Res., 67, 4427–4440.

[jgrb51169-bib-0003] Barroul, G. , and H. Kern (1996), Seismic anisotropy and shear‐wave splitting in lower‐crustal and upper‐mantle rocks from the Ivrea Zone Experimental and calculated data, Phys. Earth Planet. Inter., 95, 175–194.

[jgrb51169-bib-0004] Bianchi, I. , and G. Bokelmann (2014), Seismic signature of the alpine indentation, evidence from the Eastern Alps, J. Geodyn., 82, 69–77, doi:10.1016/j.jog.2014.07.005. 26525181 10.1016/j.jog.2014.07.005 PMC4599446

[jgrb51169-bib-0005] Bianchi, I. , N. Piana Agostinetti , C. Chiarabba , and P. De Gori (2008), Deep structure of the Colli Albani volcanic district (central Italy) from receiver functions analysis, J. Geophys. Res., 113, B09313, doi:10.1029/2007JB005548.

[jgrb51169-bib-0006] Bianchi, I. , J. Park , N. Piana Agostinetti , and V. Levin (2010), Mapping seismic anisotropy using harmonic decomposition of receiver functions: An application to Northern Apennines, Italy, J. Geophys. Res., 115, B12317, doi:10.1029/2009JB007061.

[jgrb51169-bib-0007] Boness, N. L. , and M. D. Zoback (2006), Mapping stress and structurally controlled crustal shear velocity anisotropy in California, Geology, 34(19), 825–828.

[jgrb51169-bib-0008] Chen, Y. , J. Badal , and Z. J. Zhang (2009), Radial anisotropy in the crust and upper mantle beneath the Qinghai‐Tibet Plateau and surrounding regions, J. Asian Earth Sci., 36, 289–302.

[jgrb51169-bib-0009] Crampin, S. , D. C. Booth , M. A. Krasnova , E. M. Chesnokov , and A. B. Tarasov (1986), Shear wave polarizations in the Peter the First Range indicating crack‐induced anisotropy in a thrust‐fault regime, Geophys. J. R. Astron. Soc., 84, 401–412.

[jgrb51169-bib-0010] DeMets, C. , R. G. Gordon , D. F. Argus , and S. Stein (1994), Effect of recent revisions to the geomagnetic reversal time scale on estimate of current plate motions, Geophys. Res. Lett., 21, 2191–2194, doi:10.1029/94GL02118.

[jgrb51169-bib-0011] Eckhardt, C. , and W. Rabbel (2011), P‐receiver functions of anisotropic continental crust: A hierarchic catalogue of crustal models and azimuthal waveform patterns, Geophys. J. Int., 187(1), 439–479.

[jgrb51169-bib-0012] Farra, V. , and L. Vinnik (2000), Upper mantle stratification by P and S receiver functions, Geophys. J. Int., 141(3), 699–712.

[jgrb51169-bib-0013] Frederiksen, A. W. , and M. G. Bostock (2000), Modeling teleseismic waves in dipping anisotropic structures, Geophys. J. Int., 141, 401–412.

[jgrb51169-bib-0014] Frederiksen, A. W. , H. Folsom , and G. Zandt (2003), Neighborhood inversion of teleseismic Ps conversions for anisotropy and layer dip, Geophys. J. Int., 155, 200–212.

[jgrb51169-bib-0015] Gebrande, H. (1982), Elastic wave velocities and constants of rocks and rock forming minerals, in Physical Properties of Rocks. Numerical Data and Functional Relationships in Science and Technology, edited by Angeheister G., pp. 1–96, Springer, Berlin.

[jgrb51169-bib-0016] Girardin, N. , and V. Farra (1998), Azimuthal anisotropy in the upper mantle from observation of P‐to‐S converted phases: Application to southeast Australia, Geophys. J. Int., 133, 615–629.

[jgrb51169-bib-0017] Hasegawa, A. , J. Nakajima , N. Uchida , T. Okada , D. Zhao , T. Matsuzawa , and N. Umino (2009), Plate subduction, and generation of earthquakes and magmas in Japan as inferred from seismic observations: An overview, Gondwana Res., 16, 370–400.

[jgrb51169-bib-0018] Heidbach, O. , M. Tingay , A. Barth , J. Reinecker , D. Kurfe , and B. Müller (2008), The World stress map database release.

[jgrb51169-bib-0019] Huang, Z. , D. Zhao , and L. Wang (2011a), Shear wave anisotropy in the crust, mantle wedge, and subducting Pacific slab under northeast Japan, Geochem. Geophys. Geosyst., 12, Q01002, doi:10.1029/2010GC003343.

[jgrb51169-bib-0020] Huang, Z. , D. Zhao , and L. Wang (2011b), Frequency‐dependent shear wave splitting and multilayer anisotropy in northeast Japan, Geophys. Res. Lett., 38, L08302, doi:10.1029/2011GL046804.

[jgrb51169-bib-0021] Ishise, M. , and H. Oda (2005), Three‐dimensional structure of P‐wave anisotropy beneath the Tohoku district, northeast Japan, J. Geophys. Res., 110, B07304, doi:10.1029/2004JB003599.

[jgrb51169-bib-0022] Kaneshima, S. (1990), Origin of crustal anisotropy: Shear wave splitting studies in Japan, J. Geophys. Res., 95(B7), 11,121–11,133, doi:10.1029/JB095iB07p11121.

[jgrb51169-bib-0023] Katsumata, A. (2010), Depth of the Moho discontinuity beneath the Japanese islands estimated by traveltime analysis, J. Geophys. Res., 115, B04303, doi:10.1029/2008JB005864.

[jgrb51169-bib-0024] Kimura, J.‐I. , R. J. Stern , and T. Yoshida (2005), Reinitiation of subduction and magmatic responses in SW Japan during Neogene time, GSA Bull., 117, 969–986.

[jgrb51169-bib-0025] Levin, V. , and J. Park (1997), P‐SH conversions in a flat‐layered medium with anisotropy of arbitrary orientation, Geophys. J. Int., 131(2), 253–266.

[jgrb51169-bib-0026] Levin, V. , and J. Park (1998), P‐SH conversions in layered media with hexagonally symmetric anisotropy: A cookbook, Pure Appl. Geophys., 151(2–4), 669–697.

[jgrb51169-bib-0027] Liu, H. , and F. Niu (2012), Estimating crustal seismic anisotropy with a joint analysis of radial and transverse receiver function data, Geophys. J. Int., 188, 144–164.

[jgrb51169-bib-0028] Loveless, J. P. , and B. J. Meade (2010), Geodetic imaging of plate motions, slip rates, and partitioning of deformation in Japan, J. Geophys. Res., 115, B02410, doi:10.1029/2008JB006248.

[jgrb51169-bib-0029] Mainprice, D. , and A. Nicolas (1989), Development of shape and lattice preferred orientations: Application to the seismic anisotropy of the lower crust, J. Struct. Geol., 11(2), 175–189.

[jgrb51169-bib-0030] Minato M. , M. Gorai , and M. Hunahashi (Eds.) (1965), The Geological Development of the Japanese Islands, Tsukiji Shokan, Tokyo.

[jgrb51169-bib-0031] Moschetti, M. P. , M. H. Ritzwoller , F. Lin , and Y. Yang (2010), Seismic evidence for widespread western‐US deep‐crustal deformation caused by extension, Nature, 464, 885–889, doi:10.1038/nature08951. 20376148 10.1038/nature08951

[jgrb51169-bib-0032] Nagaya, M. , H. Oda , H. Akazawa , and M. Ishise (2008), Receiver functions of seismic waves in layered anisotropic media: Application to the estimate of seismic anisotropy, Bull. Seismol. Soc. Am., 98(6), 2990–3006.

[jgrb51169-bib-0033] Nakajima, J. , and A. Hasegawa (2004), Shear‐wave polarization anisotropy and subduction‐induced flow in the mantle wedge of northeastern Japan, Earth Planet. Sci. Lett., 225(3–4), 365–377, doi:10.1016/j.epsl.2004.06.011.

[jgrb51169-bib-0034] Nakata, T. , and T. Imaizumi (2002), Digital Active Fault Map of Japan, p. 60, Univ. of Tokyo Press, Tokyo.

[jgrb51169-bib-0035] Obara, K. , K. Kasahara , S. Hori , and Y. Okada (2005), A densely distributed high‐sensitivity seismograph network in Japan: Hi‐net by National Research Institute for Earth Science Disaster Prevention, Rev. Sci. Instrum., 76, 021301.

[jgrb51169-bib-0036] Okada, T. , N. Umino , and A. Hasegawa (2008), Imaging inhomogeneous seismic velocity structure in and around the fault plane of the 2008 Iwate‐Miyagi, Japan, Nairiku earthquake (M7.2)—Spatial variation in depth of seismic‐aseismic transition and possible high‐T/overpressurized fluid distribution, AGU Fall Meet. Suppl., 89(53).

[jgrb51169-bib-0037] Okaya, D. A. , and N. I. Christensen (2002), Anisotropic effects of non‐axial seismic wave propagation in foliated crustal rocks, Geophys. Res. Lett., 29(11), 1507, doi:10.1029/2001GL014285.

[jgrb51169-bib-0038] Okaya, D. A. , and T. V. McEvilly (2003), Elastic wave propagation in anisotropic crustal material possessing arbitrary internal tilt, Geophys. J. Int., 153, 344–358.

[jgrb51169-bib-0039] Okaya, D. A. , N. I. Christensen , D. Stanley , and T. Stern (1995), Crustal anisotropy in the vicinity of the Alpine Fault Zone, South Island, New Zealand, N. Z. J. Geol. Geophys., 38, 579–583.

[jgrb51169-bib-0040] Ozacar, A. A. , and G. Zandt (2009), Crustal structure and seismic anisotropy near the San Andreas fault at Parkfield, California, Geophys. J. Int., 178, 1098–1104.

[jgrb51169-bib-0041] Park, J. J. , and V. Levin (2000), Receiver functions from multiple‐taper spectral correlation estimates, Bull. Seismol. Soc. Am., 90, 1507–1520.

[jgrb51169-bib-0042] Porter, R. , G. Zandt , and N. McQuarrie (2011), Pervasive lower‐crustal anisotropy in Southern California: Evidence for underplated schists and active tectonics, Lithosphere, 3, 201–220.

[jgrb51169-bib-0043] Rabbel, W. , and E. Lüschen (1996), Shear wave anisotropy of laminated lower crust at the Urach geothermal anomaly, Tectonophysics, 264, 219–233.

[jgrb51169-bib-0044] Rabbel, W. , and W. D. Mooney (1996), Seismic anisotropy of the crystalline crust: What does it tell us?, Terra Nova, 8, 16–21.

[jgrb51169-bib-0045] Rabbel, W. , S. Siegesmund , T. Weiss , M. Pohl , and T. Bohlen (1998), Shear wave anisotropy of laminated lower crust beneath Uarch (SW Germany): A comparison with xenoliths and with exposed lower crustal sections, Tectonophysics, 298, 337–356.

[jgrb51169-bib-0046] Sambridge, M. (1999), Geophysical inversion with a neighbourhood algorithm—I. Searching a parameter space, Geophys. J. Int., 138, 479–494.

[jgrb51169-bib-0047] Sato, H. (1994), The relationship between late Cenozoic tectonic events and stress field and basin development in northeast Japan, J. Geophys. Res., 99, 261–22.

[jgrb51169-bib-0048] Sato, H. , and K. Amano (1991), Relationship between tectonics, volcanism, sedimentation and basin development, Late Cenozoic, central part of Northern Honshu, Japan, Sediment. Geol., 74, 323–343.

[jgrb51169-bib-0049] Sato, T. , S. Miura , K. Tachibana , Y. Satake , and A. Hasegawa (2002), Crustal deformation around Ou Backbone Range, northeastern Japan observed by dense GPS network, J. Seismol. Soc. Jpn., 55, 181–191.

[jgrb51169-bib-0050] Savage, M. (1998), Lower crustal anisotropy or dipping boundaries? Effects on receiver functions and a case of study in New Zealand, J. Geophys. Res., 103(15), 69–87.

[jgrb51169-bib-0051] Savage, M. (1999), Seismic anisotropy and mantle deformation: What have we learned from shear wave splitting?, Rev. Geophys., 37, 165–206.

[jgrb51169-bib-0052] Savage, M. , J. Park , and H. Todd (2007), Velocity and anisotropy structure at the Hikurangi subduction margin, New Zealand from receiver functions, Geophys. J. Int., 168, 1034–1050.

[jgrb51169-bib-0053] Schulte‐Pelkum, V. , and K. H. Mahan (2014), A method for mapping crustal deformation and anisotropy with receiver functions and first results from {USArray}, Earth Planet. Sci. Lett., 402, 221–233, doi:10.1016/j.epsl.2014.01.050.

[jgrb51169-bib-0054] Shapiro, N. M. , M. H. Ritzwoller , P. Molnar , and V. Levin (2004), Thinning and flow of Tibetan crust constrained by seismic anisotropy, Science, 305, 233–236. 15247475 10.1126/science.1098276

[jgrb51169-bib-0055] Sherrington, H. F. , G. Zandt , and A. Frederiksen (2004), Crustal fabric in the Tibetan Plateau based on waveform inversion for seismic anisotropy parameters, J. Geophys. Res., 109, B02312, doi:10.1029/2002JB002345.

[jgrb51169-bib-0056] Sibson, R. H. (2009), Rupturing in overpressured crust during compressional inversion the case from NE Honshu, Japan, Tectonophysics, 473, 404–416.

[jgrb51169-bib-0057] Tarduno, J. A. , et al. (2003), The Emperor seamounts: Southward motion of the Hawaiian hotspot plume in Earth's mantle, Science, 301(5636), 1064–1069, doi:10.1126/science.1086442. 12881572 10.1126/science.1086442

[jgrb51169-bib-0058] Wang, Z. , W. Huang , D. Zhao , and S. Pei (2012), Mapping the Tohoku forearc: Implications for the mechanism of the 2011 East Japan earthquake (Mw 9.0), Tectonophysics, 524‐525, 147–154.

[jgrb51169-bib-0059] Watanabe, M. , and H. Oda (2014), Regional variations of the shear‐wave polarization anisotropy in the crust and mantle wedge beneath the Tohoku district, Phys. Earth Planet. Inter., 235, 49–65, doi:10.1016/j.pepi.2014.07.009.

[jgrb51169-bib-0060] Weiss, T. , S. Siegesmund , W. Rabbel , T. Bohlen , and M. Pohl (1999), Seismic velocities and anisotropy of the lower continental crust: A review, Pure Appl. Geophys., 156, 97–122.

[jgrb51169-bib-0061] Wessel, P. , and W. H. F. Smith (1998), New, improved version of the generic mapping tools released, Eos Trans. AGU, 79(47), 579–579, doi:10.1029/98EO00426.

[jgrb51169-bib-0062] Wirth, E. A. , and M. D. Long (2012), Multiple layers of seismic anisotropy and a low‐velocity region in the mantle wedge beneath Japan: Evidence from teleseismic receiver functions, Geochem. Geophys. Geosyst., 13, Q08005, doi:10.1029/2012GC004180.

[jgrb51169-bib-0063] Xia, S. , D. Zhao , X. Qiu , J. Nakajima , T. Matsuzawa , and A. Hasegawa (2007), Mapping the crustal structure under active volcanoes in central Tohoku, Japan using P and PmP data, Geophys. Res. Lett., 34, L10309, doi:10.1029/2007GL030026.

[jgrb51169-bib-0064] Yoshida, K. , A. Hasegawa , T. Okada , T. Iinuma , Y. Ito , and Y. Asano (2012), Stress before and after the 2011 great Tohoku‐oki earthquake and induced earthquakes in inland areas of eastern Japan, Geophys. Res. Lett., 39, L03302, doi:10.1029/2011GL049729.

[jgrb51169-bib-0065] Zhao, D. , S. Horiuchi , and A. Hasegawa (1992), Seismic velocity structure of the crust beneath the Japan Islands, Tectonophysics, 212, 289–301.

